# Evaluation of the antibacterial activity, surface microhardness, and color change of glass ionomer cement containing green synthesized Ag@MoS_2_ nanocomposite after thermal aging

**DOI:** 10.1007/s00784-025-06437-8

**Published:** 2025-06-25

**Authors:** Semiha Ekrikaya, Ozcan Karatas, Ebubekir Yilmaz, Nilay Ildiz, Seyma Dadi, Derya Gamze Sahin, Melih Ucar, Ismail Ocsoy

**Affiliations:** 1 Nimet Bayraktar Oral and Dental Health Center, Kayseri, Turkey; 2 Faculty of Dentistry, Department of Restorative Dentistry, Nuh Naci Yazgan University, Kayseri, Turkey; 3 Medical Imaging Department, Vocational School of Health Services, Bandirma Onyedi Eylul University, Bandirma, Turkey; 4 Department of Engineering Fundamental Sciences, Faculty of Engineering and Natural Sciences, Sivas University of Science and Technology, Sivas, Turkey; 5 Faculty of Dentistry, Nuh Naci Yazgan University, Kayseri, Turkey; 6 Faculty of Dentistry, Erciyes University, Kayseri, Turkey; 7 Department of Analytical Chemistry, Faculty of Pharmacy, Erciyes University, Kayseri, Turkey

**Keywords:** Ag@MoS_2_ nanocomposite, Antibacterial nanoparticles, Glass ionomer cement, Surface microhardness

## Abstract

**Objective:**

The aim of our study is to compare the antibacterial activity, surface microhardness, and color change of glass ionomer cement (GIC) with the addition of green-synthesized silver nanoparticles (Ag NP) and silver molybdenum disulfide (Ag@MoS_2_) nanocomposites (NC) after thermal aging conditions.

**Methods:**

Our study consisted of five groups (*n* = 7): only GIC (control); GIC + Green-synthesized Ag NP; GIC + Green-synthesized Ag@MoS_2_ NC; GIC + Chemically synthesized Ag NP, and GIC + Chemically synthesized Ag@MoS_2_ NC. The nanoparticles were synthesized, characterized using the required protocols, and added to the liquid part of the GIC. Disk-shaped samples with a diameter of 10 mm and a thickness of 2 mm were prepared with the nanoparticle-mixed GIC and used to determine the *S. mutans* live/dead assay analysis, MTT metabolic activity test, agar disk diffusion test, lactic acid production, and CFUs values. The microhardness and color change of the samples were evaluated, and statistical analysis was performed (α = 0.05).

**Results:**

Statistically significant differences were observed between the experimental and the control groups regarding live bacteria ratio, lactic acid production, inhibition zone, CFUs, and S. mutans metabolic activity (*p* < 0.05). The highest antibacterial efficacy in the experimental groups was observed in the Ag@MoS_2_ NC groups. The addition of Ag NP and Ag@MoS_2_ NC synthesized by green and chemical methods did not adversely affect the microhardness or cause clinically significant changes in the color of the experimental GICs (*p* > 0.05). Thermal aging did not adversely affect the obtained results.

**Conclusion:**

Adding Ag NP and Ag@MoS_2_ NC to GICs may provide sufficient antibacterial efficacy without adversely affecting color or microhardness values even after thermal aging.

## Introduction

Dental caries are formed by the demineralization process over time and are caused by cariogenic bacteria and fermentable carbohydrates on tooth surfaces. Acidogenic polymicrobial communities in the oral cavity are the primary source of dental caries on various surfaces, including teeth, restorative materials, and soft tissues. *Streptococcus mutans* (*S. mutans*) is known to be the most prominent species among the acidogenic bacteria contributing to caries formation. It has been observed that bacterial biofilms accumulate more on the surfaces of dental restorative materials compared to natural tooth enamel [1]. 

The biofilm content on restorative material surfaces may vary depending on the type of restorative material used. Biofilm formation can lead to surface degradation, roughening, and secondary caries, especially in restorations such as composites and glass ionomer cement (GIC) [1, 2]. Preventing secondary caries formation in restorations enhances the long-term success of the restoration, increasing patient satisfaction while reducing labor losses and financial costs [3]. Research on incorporating antibacterial properties into dental restorative materials is promising. However, such studies also reveal some limitations regarding antibacterial materials. For instance, releasable components such as chlorhexidine and benzalkonium chloride exhibit antibacterial effects when integrated into restorative materials but significantly decrease release rates over a short period [4]. 

GICs are frequently used in clinical practice and are known for their high ion release rates to help prevent caries formation. These materials offer several positive attributes, including the ability to bond to dental tissues and metal surfaces, fluoride release, compatibility with tooth structure, and biocompatibility [4]. GICs are restorative materials capable of forming chemical bonds through reactions between the phosphate ions in dental tissues and the carboxylate groups on the GIC. Some studies suggest that GICs may exhibit cariostatic effects due to the release of F- ions, while other research indicates that the F- release is below the level required for antimicrobial efficacy [4, 5]. In either case, biofilm formation can still occur on GICs, leading to the development of secondary caries at the tooth-restoration interface [1]. Therefore, it is noted that the release of F- alone is insufficient for effective antibiofilm activity on GICs. In addition to F- release, incorporating another antibacterial agent into GICs could yield positive effects without causing leakage, discoloration, or reducing mechanical strength.

While the incorporation of antibacterial agents into restorative materials has demonstrated the potential to reduce localized bacterial colonization, their overall clinical efficacy in preventing or controlling dental caries remains inconclusive. This is primarily due to the multifactorial etiology of caries, which encompasses host-related factors such as saliva composition and flow, dietary habits, oral hygiene practices, and the dynamic nature of the oral microbiome [4, 5]. Therefore, any antibacterial effect observed in vitro should be interpreted with caution, as it may not necessarily translate into meaningful long-term outcomes in vivo. Comprehensive clinical trials and longitudinal studies are required to establish the real-world therapeutic value of such antibacterial modifications.

Nanotechnology is actively utilized in many areas, including antibacterial efficacy. Nanotechnological approaches develop antibacterial materials to prevent secondary caries in restorative and preventive dentistry. Studies in this field have shown that silver nanoparticles (Ag NP) can effectively have antibacterial effects on dental materials [6]. The antimicrobial effect in dental materials containing Ag NP is based on the release of Ag ions, although the release rate of Ag NP may vary over time. This process may involve an initial intense release followed by a longer and relatively lower release of Ag NP. In recent years, it has been determined that nanocomposites (NC) obtained by adding molybdenum disulfide (MoS_2_) to the Ag NP structure (Ag@MoS_2_) may also increase the antimicrobial activity of restorative materials [7]. 

There are limited studies in the literature examining the effect of nanoparticle addition to GICs on their antibacterial activity and surface properties. In addition, comparing the effectiveness of chemically synthesized and green synthesis nanoparticles is also an important parameter. This study evaluates the antibacterial activity, mechanical, and aesthetic properties of GIC containing green synthesized nanoparticles after thermal aging. The hypotheses tested in this study are as follows:

1. No decrease in antimicrobial activity will be observed in the GIC groups containing Ag@MoS_2_ NC or Ag NP produced by green and chemical synthesis methods after thermal aging.
2. GIC containing biogenic Ag@MoS_2_ NC or Ag NP produced by the green synthesis method will exhibit more significant antibacterial effects than GIC containing Ag@MoS_2_ NC or Ag NP produced by the chemical synthesis method.
3. GIC containing biogenic Ag@MoS_2_ NC produced by green and chemical synthesis methods will exhibit more significant antibacterial effects than GIC containing Ag NP produced by these methods.
4. Adding Ag@MoS_2_ NC or Ag NP produced by green and chemical synthesis methods to GIC will not adversely affect color, aesthetic properties, or microhardness.

## Materials and methods

For the green synthesis in our study, we opted for green tea (*Camellia sinensis*) extract, which has been employed in similar previous studies [8]. 

### Preparation of green tea (*Camellia sinensis*) extract

The green tea extract was prepared following the methodologies described in the literature [8, 9]. Specifically, 2 g of powdered commercial green tea extract was dissolved in 100 mL of deionized water, heated, and filtered through Whatman No. 1 filter paper. The resulting extract was stored in a refrigerator at + 4 °C to synthesize Ag NPs, which served as both a reducing and stabilizing agent.

### Synthesis of biogenic ag NPs using green tea extract

**Fig. 1:**
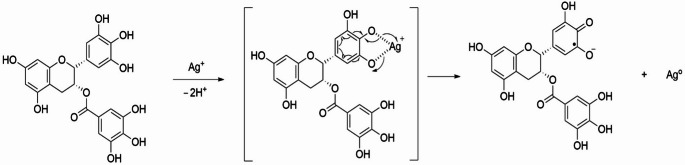
The reaction mechanism of epigallocatechin-3-gallate (EGCG) with Ag^+^ ions, leading to the reduction of Ag^+^ to Ag^0^ and the synthesis of Ag NPs

Due to its epigallocatechin-3-gallate (EGCG) content, green tea extract exhibits substantial reducing and stabilizing properties (Fig. 1). To synthesize Ag NPs, 5% green tea extract was added to a silver nitrate (AgNO_3_) solution (1 mM, 50 mL) and incubated (WiseBath WSB-18, Daihan Scientific, Korea) at 85 °C for 1 h under magnetic stirring. After incubation, the mixture was cooled to room temperature and centrifuged at 12,000 rpm for 10 min to remove the extract from the medium. The residue was washed with deionized water and centrifuged again (Universal 320 R, Hettich Zentrifugen, Tuttlingen, Germany). The excess plant extract was removed with the supernatant. The resulting Ag NP solutions were stored at + 4 °C for characterization [9]. The absorbance spectra of Ag NPs and green tea extract were recorded using a UV-Vis spectrophotometer.

### Chemical synthesis of ag NPs

The Turkevich method was employed for the chemical synthesis of Ag NPs [10]. A 7 mM trisodium citrate solution (100 mL) was prepared, and the pH was adjusted to 8 using NaOH. The solution was brought to a boil under magnetic stirring, followed by adding 1 mL of 1 mM AgNO_3_ solution. The solution’s color changed from yellow to light brown over time, indicating the formation of Ag NPs. After cooling to room temperature, the mixture was centrifuged at 12,000 rpm for 10 min. The synthesized Ag NPs were stored at + 4 °C for further characterization and applications.

### Synthesis of Ag@MoS_2_ nanocomposite

The synthesis of Ag@MoS_2_ was carried out by modifying a technique described in the literature [11]. Commercially obtained MoS_2_ nanosheets (MoS₂ nanosheets were purchased from Sigma-Aldrich (Product No. 69860, St. Louis, MO, USA)) were combined with Ag NPs in two stages, using different concentrations of NaCl solution to facilitate the binding. Initially, Ag NPs were added to MoS_2_ dispersed homogeneously in deionized water (0.1 mg/mL). Subsequently, NaCl solution (2.4 mL, 0.09 M) was added dropwise, and the mixture was continuously stirred. In the second stage, an additional NaCl solution (5 mL, 0.29 M) was added dropwise. The mixture was then centrifuged at 5,000 rpm for 5 min to separate the precipitated Ag@MoS_2_ nanocomposite from the medium, which was then re-dispersed in deionized water and centrifuged again. The final Ag@MoS_2_ nanocomposite was stored at + 4 °C.

### Characterization of ag NPs and ag@MoS_2_ nanocomposite

The morphology of the chemically and green-synthesized Ag NPs and Ag@MoS_2_ nanocomposites was determined using scanning electron microscopy (SEM) (LEO-440, Zeiss, Cambridge, UK). The elemental composition of Ag NPs and the presence of Ag in the Ag@MoS_2_ nanocomposite were confirmed by energy-dispersive X-ray (EDX) spectroscopy. The hydrodynamic diameters of the Ag NPs and Ag@MoS_2_ nanocomposites were measured using dynamic light scattering (DLS), and their surface charges were determined via zeta potential (ZT) analysis. Additionally, the characteristic absorbance peak of the Ag NPs was identified using UV-Vis spectrometry. The presence of green tea extract on the surface of the Ag NPs was confirmed using Fourier-transform infrared (FT-IR) spectroscopy [8]. 

### Sample size calculation

The required sample size was estimated based on a priori power analysis, informed by data from a comparable previously published study [12]. The analysis was performed using G*Power software (version 3.1), with parameters set at an alpha level of 0.05, a statistical power (1–β) of 0.90, and a large effect size (Cohen’s d = 1.0), in accordance with conventional thresholds for behavioral and biomedical research. Among the planned statistical tests, the calculation yielding the largest sample size requirement indicated that a minimum of seven subjects (*n* = 7) would be sufficient to detect a statistically significant effect. These parameters were selected to ensure adequate power to detect meaningful differences while minimizing the risk of Type II errors.

### Formation of study groups

The study included five groups, comprising seven samples (*n* = 7). A commercial GIC (Fuji IX, GC Corporation, Tokyo, Japan) was used as the base material, and samples were tested before and after thermal aging equivalent to 12 months (3600 thermal cycles):

1. GIC (control group).
2. GIC + Green synthesized Ag NPs.
3. GIC + Green synthesized Ag@MoS_2_ nanocomposite.
4. GIC + Chemically synthesized Ag NPs.
5. GIC + Chemically synthesized Ag@MoS_2_ nanocomposite.

### Preparation of experimental GIC cements and samples

The mixing method used to prepare the experimental GIC was selected by taking a similar study as a reference and checking the homogeneous distribution of Ag NPs with EDX mapping [4]. The samples obtained a more homogeneous distribution When Ag NPs were added to the GIC liquid phase. The synthesized NPs were incorporated into commercial restorative glass ionomer cement (Fuji IX) at 0.025%, 0.05%, 0.10%, and 0.50% by weight. Minimum Inhibitory Concentration (MIC) test was used to determine the sufficient concentration providing antibacterial activity. As a result of the test, 0.05%, the lowest concentration providing antimicrobial activity, was selected. Ag NPs were added to the liquid phase of each GIC capsule by removing the capsule piston. After the piston was reattached, activation was initiated by pushing, and the mixture was mixed in a high-speed amalgamator for the time recommended by the GIC manufacturer (10 s). The experimental GICs were placed in round silicone molds (10 mm diameter, 2 mm thickness) with an application gun and adapted with a spatula to obtain disc-shaped samples. They were pressed with glass and waited 6 min for setting reaction according to the manufacturer’s instructions. Another 24 h were waited for setting reaction. The samples designed for antibacterial tests and microhardness tests were polished under continuous water flow using rotary tools and abrasive disks with increasing grit sizes (1000, 1500, and 2000 grit) and then rinsed with distilled water. Samples intended for antibacterial tests were sterilized with ethylene oxide (% 100 ethylene oxide).

### EDX analysis

Energy dispersive X-ray (EDX) spectroscopy was used to determine the concentration and homogeneous distribution of Ag NPs in the Ag@MoS_2_ nanocomposite. Additionally, the concentration and homogeneous distribution of Ag NPs in the experimental GIC prepared polymerized samples containing Ag@MoS_2_ were determined using EDX mapping.

### Thermal aging

Thermal aging of the samples was performed similarly to a previous study [12]. Thermal cycling was conducted at 5 °C and 65 °C, with 30 s of immersion and 10-second intervals. The samples underwent 3600 cycles of thermal aging, equivalent to approximately 12 months of oral exposure. Non-thermally aged samples were stored in distilled water at 37 °C. After thermal aging, all samples were stored in distilled water at 37 °C for 24 h before the relevant tests were conducted.

### Surface microhardness test

Seven disk-shaped samples from each group were prepared and polished for the surface microhardness test. The test used a digital microhardness tester (DuraScan-20, Struers, Denmark). The measurements were recorded as Vickers hardness number (VHN) (kgf/mm^2^). The indentation required for determining the composite surface hardness was performed by applying a force of 100 gf for 15 s with a 10 μm diameter tip. Measurements were taken from 8 points on the surface of each composite sample by changing the sample clockwise, and the average of these values ​​was recorded [13]. 

### Color measurement of GIC

Color changes in the experimental GIC were measured using the VITA Easyshade^®^ V spectrophotometer (VITA Easyshade V, Vita Zahnfabrik, Bad Sackingen, Germany) and calculated using the CIE L*a*b* color system with the following formula:

∆E = [(∆L*) ^2^ + (∆a*) ^2^ + (∆b*) ^2^] ^1/2^.

GIC disks (10 mm diameter × 2 mm thickness) were prepared using silicone molds for the measurements (*n* = 7). The initial color of all GIC samples was selected as A2. Color measurements were taken immediately after preparation and after 3600 thermal cycles, with the color change relative to the initial color being recorded [14]. 

### Antimicrobial tests

*Streptococcus mutans (S. mutans)* ATCC 25,175 strain was used for antimicrobial tests. To develop a 24-hour *S. mutans* biofilm, CIS specimens were positioned in 24-well plates. A saliva-glycerol stock solution was diluted 1:50 in either BHI or McBain medium, and 1.5 mL of this inoculum was added to each well. The samples were incubated at 37 °C in a 5% CO₂ atmosphere for 8 h. Following this initial incubation, the discs were transferred to fresh 24-well plates and subjected to a further 16-hour incubation under identical conditions. Subsequently, the discs were placed into new 24-well plates once more and incubated for an additional 24 h at 37 °C in a 5% CO₂ environment to allow for mature biofilm formation. 24 All analyses were repeated for 7 samples from each group.

#### Live/Dead bacterial assay

Following rinsing with phosphate-buffered saline (PBS), the biofilm-coated discs were stained using the Live/Dead BacLight Bacterial Viability Kit (Molecular Probes, Eugene, OR). SYTO 9 selectively stained viable bacteria with intact membranes, emitting green fluorescence, whereas propidium iodide (PI) penetrated compromised membranes, marking dead or damaged cells with red fluorescence. The stained biofilms were subsequently imaged using a confocal laser scanning microscope (Zeiss LSM 710, Germany) [15–17]. 

#### *S. mutans* metabolic activity (MTT Assay)

Discs with 24-hour *S. mutans* biofilms were transferred into a fresh 24-well plate containing 1 mL of MTT solution (0.5 mg/mL in PBS) per well. The samples were incubated at 37 °C in a 5% CO₂ atmosphere for 1 h, during which metabolically active bacteria reduced the MTT to purple formazan crystals. After incubation, each disc was transferred to a new well, and 1 mL of dimethyl sulfoxide (DMSO) was added to solubilize the formazan. Following 20 min of incubation in the dark, the DMSO extracts were transferred to a 96-well microplate, and the absorbance was measured at 540 nm (OD540) using a microplate reader (SpectraMax M5, Molecular Devices, Sunnyvale, CA) [15–17]. 

#### Agar disc diffusion test

Mueller-Hinton agar was employed as the testing medium. After autoclaving and pouring into Petri dishes, wells of 6 mm in diameter were created using a sterile cork borer. Bacterial suspensions were prepared using the standard inoculum method; specifically, a 0.5 McFarland bacterial suspension was prepared from 18–24-hour-old cultures and uniformly spread onto agar plates using a sterile cotton swab, according to EUCAST guidelines (2019). The plates were incubated under anaerobic conditions for 24 h, after which the zones of inhibition were measured. All experiments were performed in triplicate and included appropriate positive and negative controls [15–17]. 

#### Lactic acid production

To remove loosely attached bacteria, discs with 24-hour *Streptococcus mutans* biofilms were washed with cysteine peptone water (CPW). The discs were then placed in 24-well plates containing buffered peptone water (BPW) supplemented with 0.2% sucrose. Samples were incubated at 37 °C in a 5% CO₂ atmosphere for 3 h to allow acid production. The lactate concentration in the BPW supernatants was measured enzymatically, and absorbance at 340 nm was recorded using a microplate reader [16, 17]. 

#### Biofilm colony forming units (CFUs)

To quantify viable bacterial populations, tryptic soy agar supplemented with blood was used. Discs bearing 24-hour *S. mutans* biofilms were transferred into 2 mL microtubes. The biofilms were detached via sonication (3510RMTH, Branson, Danbury, CT), followed by mixing for 5 min and vortexing at 2400 rpm for 30 s (Fisher Scientific, Pittsburgh, PA). Serial dilutions of the resulting suspension were plated, and after incubation, colony counts were used to calculate the colony-forming units (CFUs) per disc, adjusting for the dilution factor [15–17]. 

### Statistical analysis

Statistical analysis of antibacterial tests was performed using One-Way ANOVA with GraphPad Prism version 8.0.1. Two-Way ANOVA was performed using SPSS software (IBM SPSS Statistics V21.0) for the microhardness test. The normal distribution of the data was examined using the Shapiro-Wilk test, and the homogeneous distribution was analyzed using the Levene test. Tukey posthoc test was used for mean comparison. Descriptive statistics are given as number of units (n), percentage (%), and mean ± standard deviation (x̄ ± ss). Statistical significance level (*p* < 0.05) was accepted.

## Results

### Synthesis and characterization of ag NP and ag@MoS_2_ NC

#### UV spectra, STEM imaging, EDX analysis of ag NP and ag@MoS_2_ NC

**Fig. 2:**
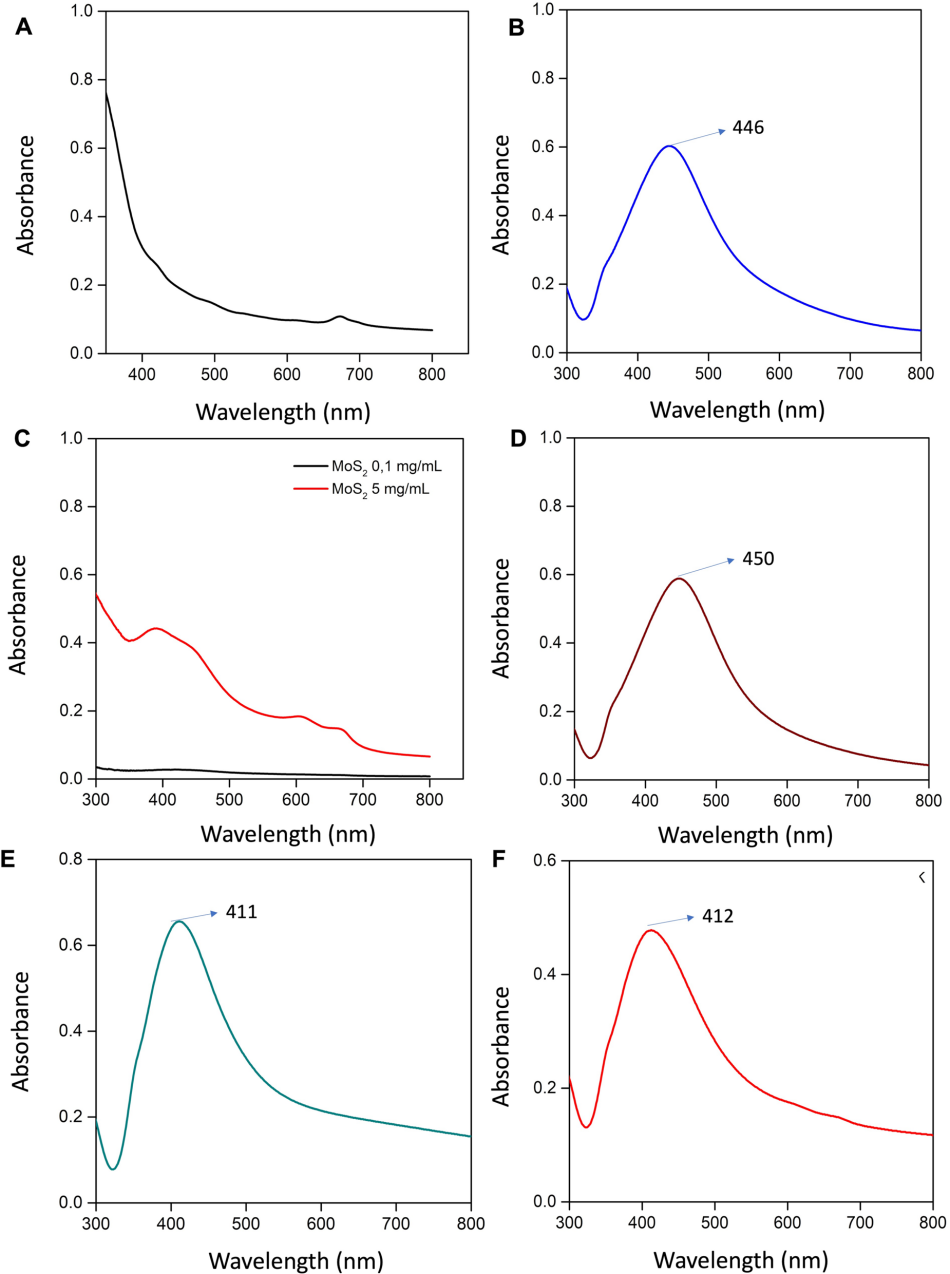
(**A**) 5% green tea extract, (**B**) UV spectrum of green (biogenic) synthesized Ag NP, (**C**) UV spectra of 0.1 mg/mL and 5 mg/mL MoS_2_ solutions, (**D**) UV spectrum of green (biogenic) synthesized Ag@MoS_2_ NC, (**E**) UV spectrum of chemically synthesized Ag NP, (**F**) UV spectrum of chemically synthesized Ag@MoS_2_ NC

**Fig. 3:**
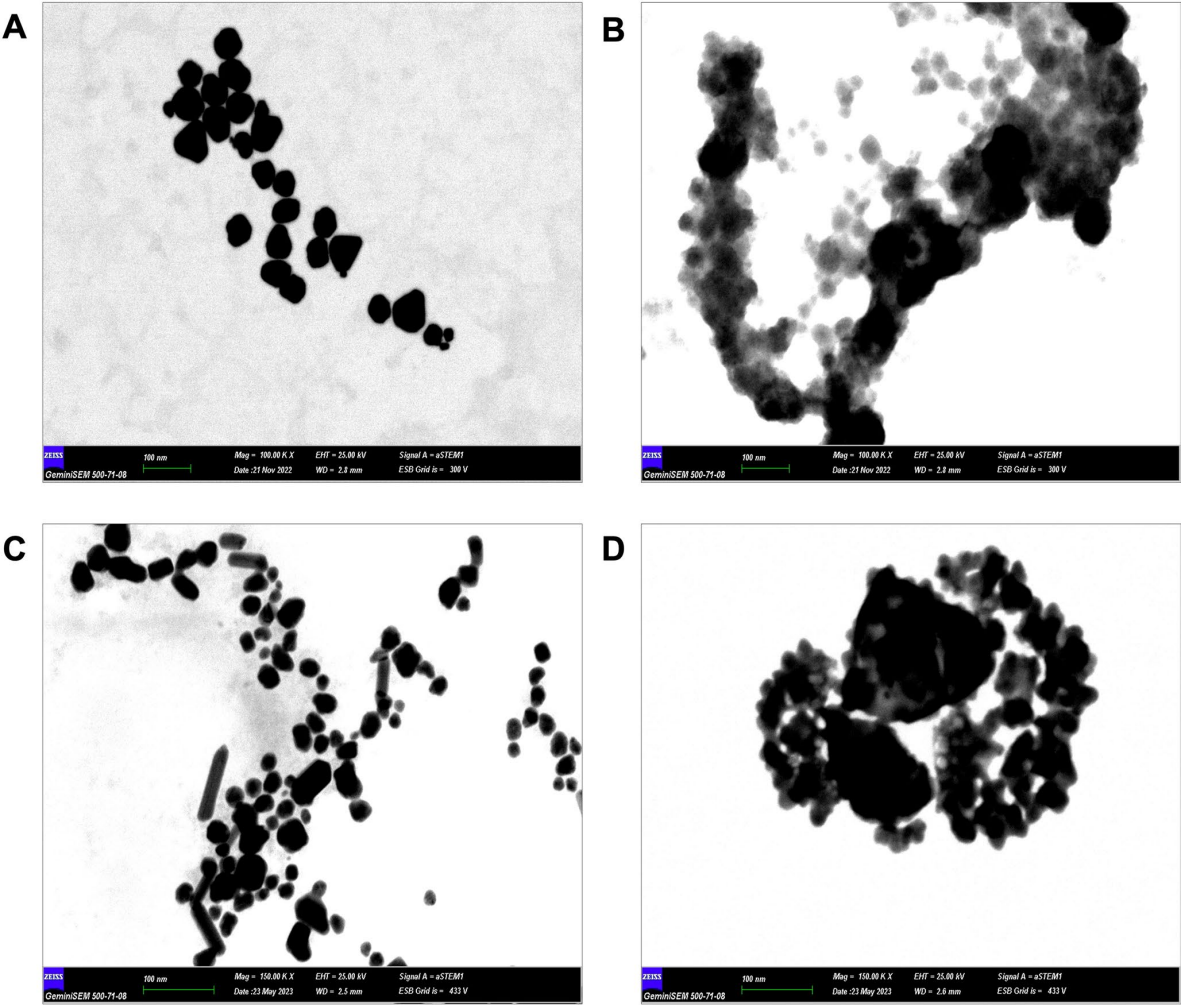
(**A**) STEM image of green (biogenic) synthesized Ag NP, (**B**) STEM image of green (biogenic) synthesized Ag@MoS_2_ NC, (**C**) STEM image of chemically synthesized Ag NP, (**D**) STEM image of chemically synthesized Ag@MoS_2_ NC

The UV spectra of Ag NPs synthesized via chemical and green methods are shown in Figs. 2 and 3. As demonstrated in Fig. 2, the UV spectrum of the synthesized NPs indicates the characteristic absorbance peaks of the green-synthesised Ag NPs and Ag@MoS_2_ NC at 446 nm and 450 nm, respectively. Furthermore, the peaks of the absorbance spectra of chemically synthesized Ag NPs and Ag@MoS_2_ NC are observed at 411 nm and 412 nm, respectively [18]. As shown in Fig. 1C, MoS_2_ did not exhibit an absorbance peak at low concentrations, and therefore, no absorbance peak attributable to MoS_2_ was detected in the UV spectra of Ag@MoS_2_ NC. Figure 3 presents STEM images of the synthesized Ag NP and Ag@MoS2 NC. The Ag NPs synthesized using both green and chemical methods exhibit a homogeneous spherical morphology with approximate dimensions of 20 nm.

**Fig. 4:**
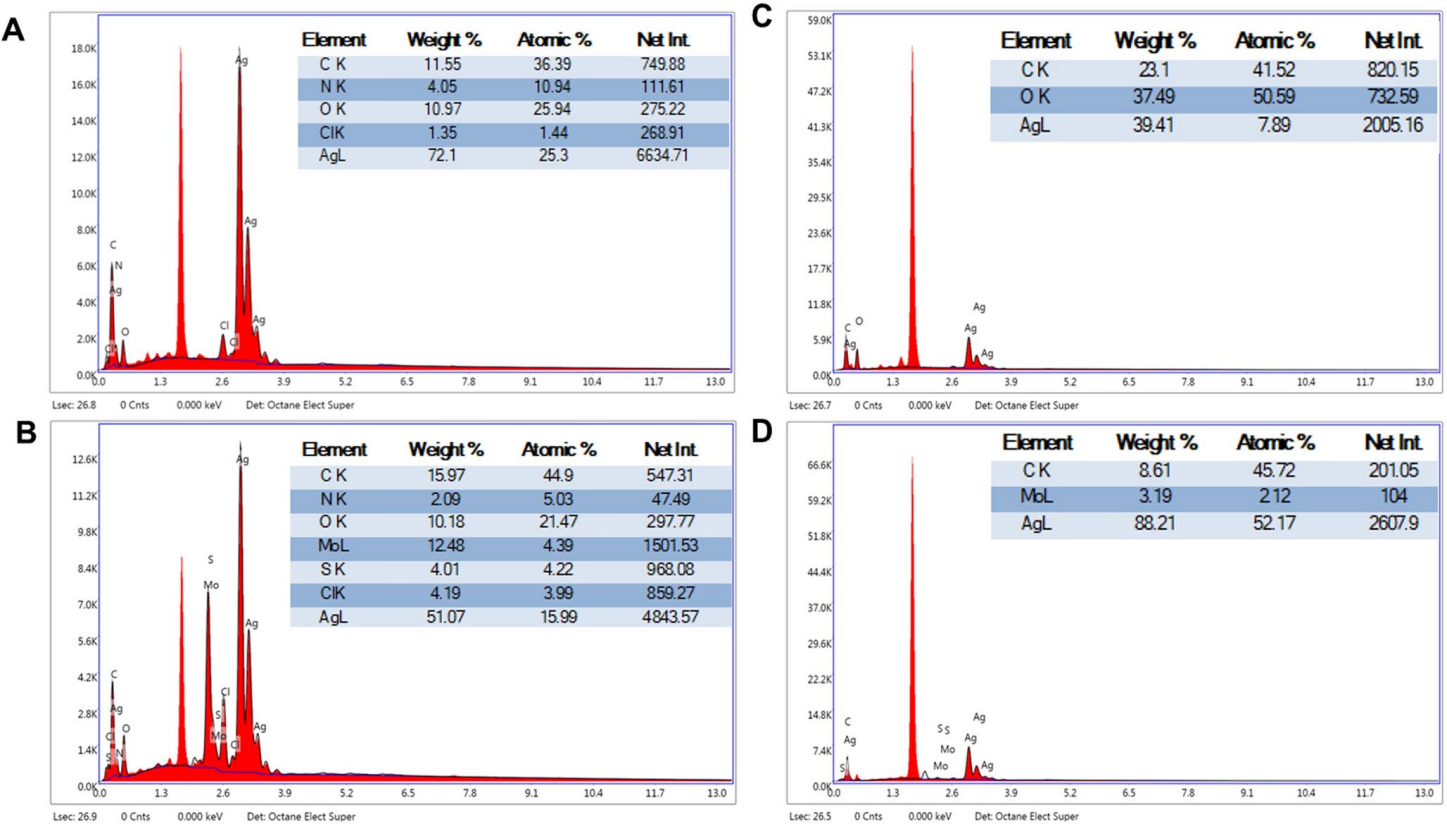
(**A**) EDX spectrum of green (biogenic) synthesized Ag NP, (**B**) EDX spectrum of green (biogenic) synthesized Ag@MoS_2_ NC, (**C**) EDX spectrum of chemically synthesized Ag NP, (D) EDX spectrum of chemically synthesized Ag@MoS_2_ NC

The presence and ratio of Ag and Mo in Ag NP and Ag@MoS_2_ NC were determined using Energy Dispersive X-ray Spectroscopy (EDX) (Fig. 4).

#### FT-IR analysis

**Fig. 5:**
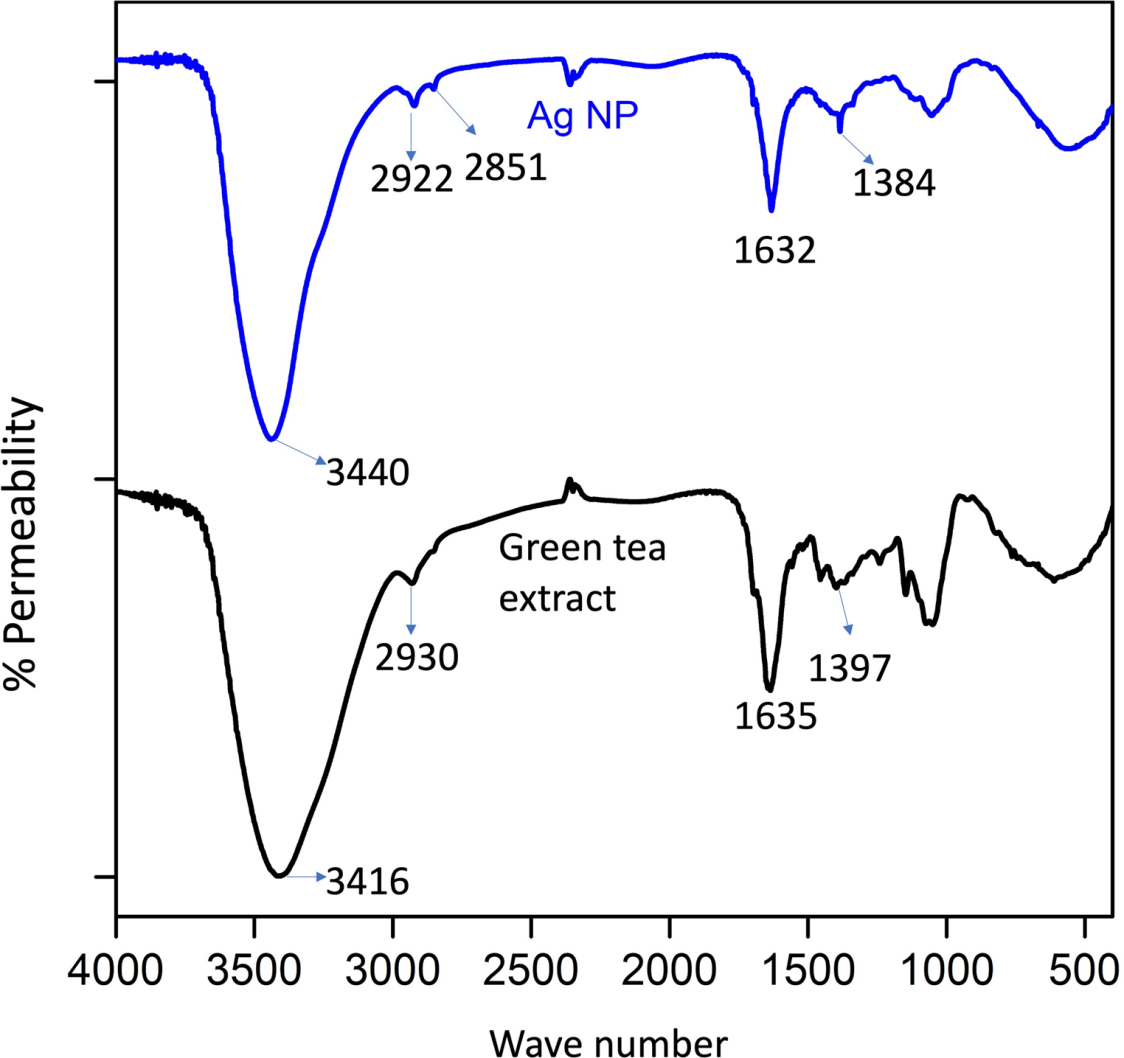
FT-IR spectra of green tea extract and green synthesized Ag NP

The presence of green tea extract in Ag NPs was elucidated through FT-IR analysis. The peak at 3416 cm⁻¹ in the green tea extract’s FT-IR spectrum corresponds to the hydroxyl groups’ stretching vibrations. In comparison, the peak at 2930 cm⁻¹ is associated with aliphatic group vibration frequencies. Additionally, the peak at 1635 cm⁻¹ indicates the stretching vibration of C = O groups in ketones, quinones, and esters, and the peak at 1397 cm⁻¹ represents the stretching vibration of C-N groups in aromatic amines. The peaks at 3440 cm⁻¹, 2922 cm⁻¹, 1632 cm⁻¹, and 1384 cm⁻¹ in the FT-IR spectrum of Ag NP indicate the presence of green tea extract on the surface of the nanoparticles (Fig. 5).

#### DLS and zeta potential of ag NP and ag@MoS_2_ NC

**Fig. 6:**
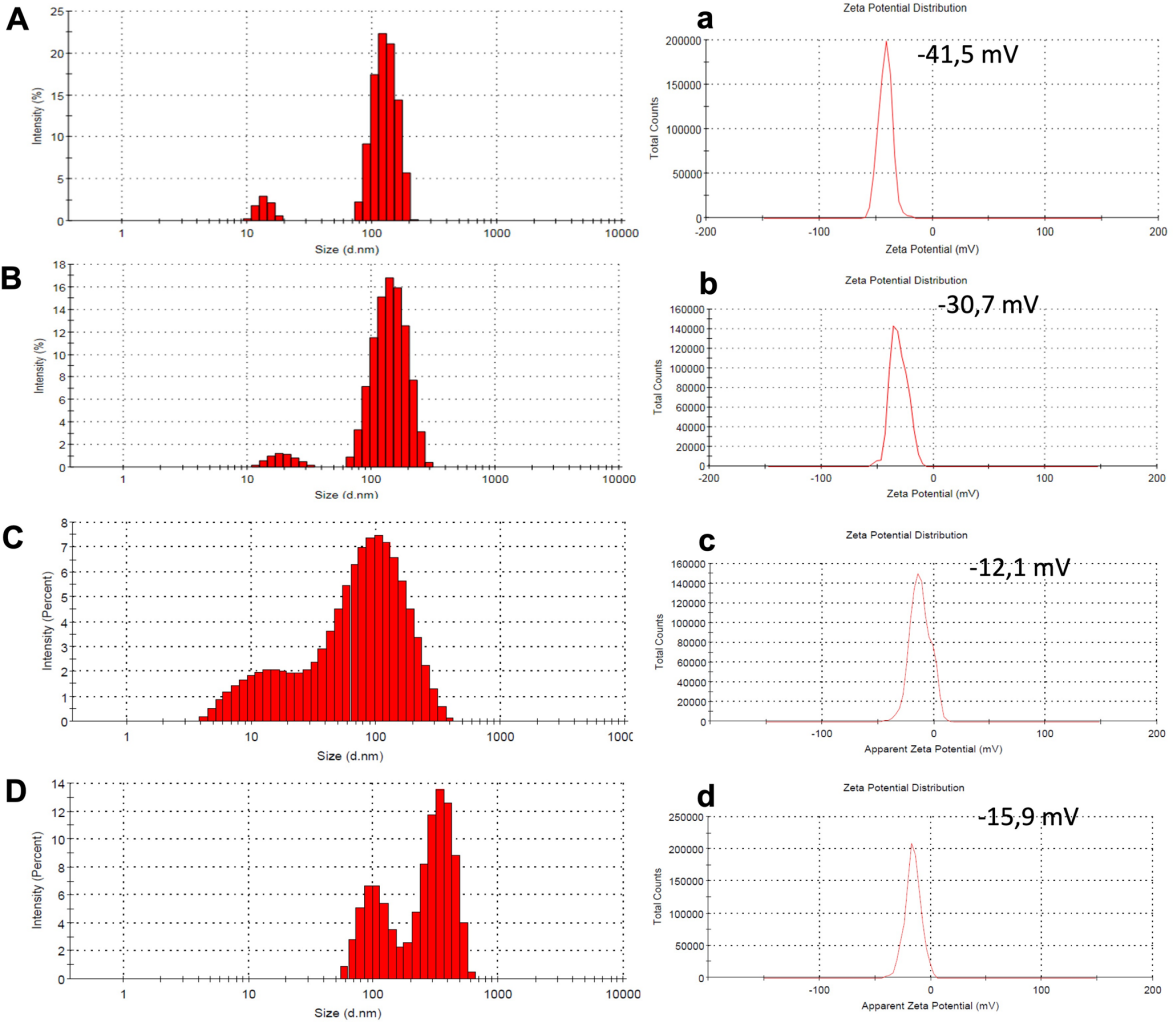
**A**) DLS analysis of green (biogenic) synthesized Ag NP, (**a**) Zeta analysis of green (biogenic) synthesized Ag NP, **B**) DLS analysis of green synthesized Ag@MoS_2_, (**b**) Zeta analysis of green synthesized Ag@MoS_2_, **C**) DLS analysis of chemically synthesized Ag NP, (**c**) Zeta analysis of chemically synthesized Ag NP, **D**) DLS analysis of chemically synthesized Ag@MoS_2_, (**d**) Zeta analysis of chemically synthesized Ag@MoS_2_

The hydrodynamic diameters of Ag NP and Ag@MoS_2_ NC were measured using Dynamic Light Scattering (DLS), while their surface charges were assessed with a zeta sizer. The DLS values for green synthesized Ag NP and Ag@MoS_2_ NC were 134 nm and 173 nm, respectively, whereas the DLS values for chemically synthesized Ag NP and Ag@MoS_2_ NC were 109 nm and 341 nm, respectively (Fig. 6). DLS measures the hydrodynamic diameter of nanoparticles, which includes not only the core size but also the surrounding layer of water molecules and any possible biomolecular coatings on the surface. Therefore, the sizes obtained by DLS are generally larger than the actual physical sizes measured by microscopy techniques such as STEM. This difference becomes more pronounced when a distinct solvation layer is present around the particles. The negative zeta potential values indicate a uniform distribution of surface charges and stability of the nanoparticles and nanocomposites in aqueous environments.

### Microhardness test

Table 1 presents the groups’ microhardness values (kgf/mm^2^). The measured values show no significant difference between the groups with different compositions or after thermal aging.

**Table 1:** Microhardness values (kgf/mm^2^) of the groups

		Minimum	Maximum	Mean ± Sd
No thermal cycling	**Control**	92,80	110,00	101,13 ± 3,00^a^
	**G- AgNP**	93,60	106,00	98,26 ± 2,92^a^
	**G - Ag@MoS**_**2**_	92,00	108,00	97,51 ± 2,70^a^
	**C - AgNP**	98,20	108,00	100,88 ± 1,69^a^
	**C - Ag@MoS**_**2**_	87,20	104,00	97,83 ± 2,39^a^
3600 thermal cycling	**Control**	96,70	108,00	98,31 ± 1,50^a^
	**G - AgNP**	89,70	108,00	99,20 ± 3,14^a^
	**G - Ag@MoS**_**2**_	97,90	109,00	96,98 ± 1,51^a^
	**C - AgNP**	90,20	107,00	97,45 ± 2,70^a^
	**C - Ag@MoS**_**2**_	89,70	104,00	97,61 ± 2,33^a^

* Different superscript letters (a, b,…,f) indicate statistical differences

**G– AgNP**: Green synthesis Ag NP, **G - Ag@MoS**_**2**_: Green synthesis Ag@MoS_2_ NC, **C– AgNP**: Chemical synthesis Ag NP, **C - Ag@MoS**_**2**_: Chemical synthesis Ag@MoS_2_ NC, **Sd**: Standard deviation

### Color change

Table 2 presents the color change values of the GIC samples based on color measurements taken at the initial stage, after adding NP and NC, and after thermal aging. The ΔE values calculated from these measurements indicate no clinically significant color change.

**Table 2:** Mean ± standard deviations of ΔE (0) and ΔE (0-TC). One-Way ANOVA was used for comparisons (*n* = 7)

Group	ΔE (0) ± Sd	ΔE (0-TC) ± Sd	Statistics Test
**Control**		1.13 ± 0.42^b^	
**G- AgNP**	1.23 ± 0.24^a^	1.56 ± 0.65^b^	p: 0.65
**G - Ag@MoS**_**2**_	1.58 ± 0.78^a^	1.67 ± 0.41^b^	p: 0.79
**C - AgNP**	1.38 ± 0.56^a^	1.69 ± 0.36^b^	p: 1.35
**C - Ag@MoS**_**2**_	1.17 ± 0.69^a^	1.75 ± 0.65^b^	p: 0.97
**Statistics Test**	p: 0.32	p: 0.58	

* Different superscript letters (a, b,…,f) indicate statistical differences

**G– Ag NP**: Green synthesis Ag NP, **G - Ag@MoS**_**2**_: Green synthesis Ag@MoS_2_ NC, **C– Ag NP**: Chemical synthesis Ag NP, **C - Ag@MoS**_**2**_: Chemical synthesis Ag@MoS_2_ NC, **Sd**: Standard deviation

ΔE < 3.3: Clinically acceptable limit. ΔE ≥ 3.3: Color difference is apparent and is considered a clinically significant color change

**ΔE (0)**: Color comparison of the control group and nanoparticle added groups before the thermal cycle, **ΔE (0-TC)**: Color comparison of the control group and nanoparticle added groups after 3600 thermal cycles (TC)

### Antibacterial tests

Table 3 gives values for all groups live bacteria, S. mutans MTT metabolic activity, agar disk diffusion, lactic acid production, and CFUs ​​.

**Table 3:** Live bacteria, S. *mutans* MTT metabolic activity, agar disk diffusion, lactic acid production and *CFUs* values ​​of the groups. One-Way ANOVA was used for comparisons (*n* = 7)

Groups	Live Bacteria (%)	S. mutans MTT activity	Agar disk diffusion (mm)	Lactic acid production	CFU_s_
**Control**	78.32 (± 0.541)^a^	0.587 (± 0.587)^a^	7.054 (± 0.123)^a^	11.425 (± 0.145)^a^	22.35 (± 0.98) ^a^
**G-Ag NP**	38.23 (± 0.325)^b^	0.315 (± 0.369)^b^	18.08 (± 0.271)^b^	9.153 (± 0.054)^b^	9.56 (± 0.42)^b^
**G-Ag@MoS**_**2**_ **NC**	31.25 (± 0.145)^c^	0.258 (± 0.214)^c^	21.07 (± 0.113)^c^	6.14 (± 0.078)^c^	7.96 (± 0.85)^c^
**C-Ag NC**	39.78 (± 0.236)^b^	0.298 (± 0.758)^b^	16.147 (± 0.178)^b^	9.939 (± 0.178)^b^	10.78 (± 0.52)^b^
**C-Ag@MoS**_**2**_ **NC**	34.36 (± 0.785)^c^	0.214 (± 0.321)^c^	20.123 (± 0.352)^c^	7.874 (± 0.178)^c^	8.7 (± 0.74)^c^
**TC-Control**	75.25 (± 0.98)^a^	0.564 (± 0541)^a^	6.987 (± 0.059)^a^	11.989 (± 0.078)^a^	21.47 (± 0.87) ^a^
**TC-G-Ag NP**	42.78 (± 0.355)^b^	0.320 (± 0.096)^b^	17.58 (± 0.278)^b^	9.658 (± 0.0147)^b^	10.180.85)^b^
**TC-G-Ag@MoS**_**2**_ **NC**	36.87 (± 0.358)^c^	0.275 (± 0.319)^c^	20.857 (± 0.785)^c^	6.87 (± 0.048)^c^	8.85 (± 0.63)^c^
**TC-C-Ag NP**	43.89 (± 0.325)^b^	0.302 (± 0.044)^b^	16.01 (± 0.241)^b^	10.437 (± 0.089)^b^	11.12 (± 0.399)^b^
**TC-C-Ag@MoS**_**2**_ **NC**	37.88 (± 0.458)^c^	0.243 (± 0.568)^c^	19.145 (± 0.132)^c^	8.065 (± 0.258)^c^	9.1 (± 0.85)^c^

* Different superscript letters (a, b,…,f) indicate statistical differences

**G– Ag NP**: Green synthesis Ag NP, **G - Ag@MoS**_**2**_: Green synthesis Ag@MoS_2_ NC, **C– Ag NP**: Chemical synthesis Ag NP, **C - Ag@MoS**_**2**_: Chemical synthesis Ag@MoS_2_ NC, **TC**: Thermal cycling

#### Live/Dead assay

**Fig. 7:**
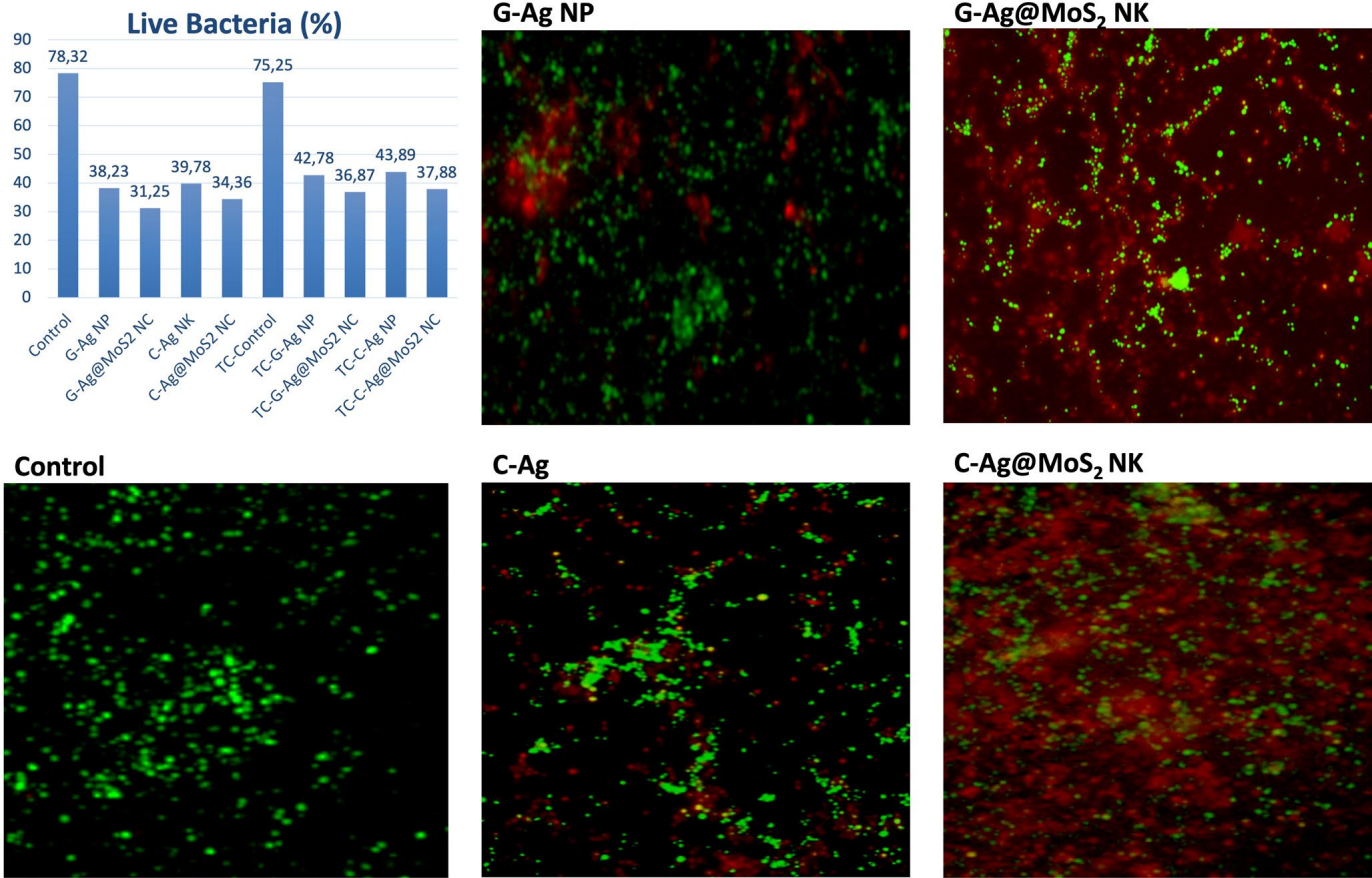
Percentage of live bacteria and confocal microscopy images. G - AgNP: Green synthesized Ag NP, G - Ag@MoS_2_: Green synthesized Ag@MoS_2_ NC, C - AgNP: Chemically synthesized Ag NP, C - Ag@MoS_2_: Chemically synthesized Ag@MoS_2_ NC, TC: Thermal cycling

A statistically significant difference was observed between the groups (*p* < 0.05). The lowest live/dead ratio of bacteria was found in both the Ag@MoS_2_ NC groups with and without thermal cycling. The highest live/dead ratio was observed in the control groups (Fig. 7).

#### *S. mutans* metabolic activity (MTT Assay)

When evaluating the metabolic activities of *S. mutans*, the highest metabolic activity was observed in the control groups, both with and without thermal cycling. The lowest metabolic activity was observed in the Ag@MoS_2_ nanocomposite (NC) groups, also with and without thermal cycling (*p* < 0.05) (Table 3).

#### Agar disk diffusion test

In terms of inhibition zones, the largest zone was observed in the Ag@MoS_2_ NC groups, both with and without thermal cycling, while the most minor inhibition zone was seen in the control groups under the same conditions (*p* < 0.05) (Table 3).

#### Lactic acid production

The highest lactic acid production was observed in the control groups, both with and without thermal cycling. In contrast, the lowest production was noted in the Ag@MoS_2_ NC groups, under both conditions (*p* < 0.05) (Table 3).

#### *S. mutans* Colony-Forming units (CFUs)

The highest number of *S. mutans* CFUs was observed in the control groups, both with and without thermal cycling. Conversely, the lowest number was recorded in the Ag@MoS_2_ NC groups, under both conditions (*p* < 0.05) (Table 3).

## Discussion

This study evaluated the antibacterial activity, surface microhardness, and color stability of experimental resin-modified glass ionomer cements (RMGICs) modified with 0.05% (w/w) silver-based nanoparticles, including biogenic silver nanoparticles (Ag NPs) and silver molybdenum disulfide nanocomposites (Ag@MoS₂ NC), synthesized via both green and chemical methods. Additionally, the influence of artificial aging through 3600 thermal cycles was assessed to simulate long-term intraoral conditions. The results demonstrated that the incorporation of both types of nanoparticles significantly enhanced the antibacterial efficacy of RMGICs compared to the control group without nanoparticles. This was evident from reductions in the number of viable Streptococcus mutans, metabolic activity, lactic acid production, and colony-forming units (CFUs), as well as increased inhibition zones in the agar diffusion assay. Notably, RMGICs containing Ag@MoS₂ NC particularly those synthesized chemically exhibited the highest antibacterial performance. Despite these enhancements, the addition of 0.05% nanoparticles did not negatively impact the surface microhardness or result in clinically significant color changes, even after thermal cycling. These findings suggest that incorporating Ag NP and Ag@MoS₂ NC into RMGICs can provide sustained antibacterial effects under simulated oral conditions without compromising the material’s structural or aesthetic properties.

Nanoparticles (NPs) can be synthesized through chemical, physical, and biological (green) methods [8, 15]. Among these, green synthesis which employs biological agents such as plant extracts, enzymes, and proteins has gained attention due to its non-toxic, biocompatible, and environmentally friendly nature. Unlike chemical methods that often involve toxic reducing and stabilizing agents, green synthesis offers a safer alternative with additional benefits such as simplicity, low cost, and rapid reaction rates [8]. Plant extracts are particularly favored in this context due to their easy availability, low contamination risk, and natural capping and reducing abilities [16]. For instance, green tea (*Camellia sinensis*) extract, rich in epigallocatechin-3-gallate (EGCG), effectively reduces silver ions (Ag⁺) to metallic silver (Ag⁰) while stabilizing the nanoparticles to prevent aggregation [8]. Studies have shown that silver nanoparticles synthesized using green tea extract exhibit significant antibacterial activity against a broad range of bacterial strains, including *Streptococcus mutans*,* Staphylococcus aureus*,* Pseudomonas aeruginosa*,* Klebsiella pneumoniae*,* Salmonella enterica*,* and Escherichia coli* [17, 18]. 

In recent years, two-dimensional nanomaterials (NMs) have been extensively researched due to their interesting physical and chemical properties and potential applications in electronics and medicine. Studies on two-dimensional nanomaterials are often derived from the well-known and frequently studied graphene [19]. Today, two-dimensional nanomaterials, especially graphene derivatives like graphene oxide (GO), are widely used as carriers to stabilize nanoparticles, prevent aggregation, and increase surface area. Ag NP fixed on the surface of GO offers greater antibacterial efficacy by increasing the surface area [13, 20, 21]. The exploration of graphene and its applications has stimulated the discovery of other two-dimensional nanomaterials, such as molybdenum disulfide [19]. In contemporary literature, various nanoparticles are loaded onto MoS_2_ for use as biosensors and antibacterial agents [22]. Additionally, MoS_2_ is actively studied for use on the surfaces of implants and orthodontic materials in dentistry due to its antibacterial properties [23]. Moreover, because GO and reduced GO (rGO) solutions are brown or black, their use in aesthetic materials is limited. In contrast, the MoS_2_ solution is gray-transparent, making it a more advantageous option for aesthetic materials.

In our study, biocompatible silver Ag NP synthesized by the green method was fixed onto MoS_2_ to produce Ag@MoS_2_ NC. Green tea extract was preferred for the green synthesis due to its component EGCG, which offers excellent reducing and stabilizing properties [8]. The literature generally uses chemical synthesis methods for Ag NP-containing GICs. In our study, the green synthesis method was employed, thus eliminating the disadvantages of chemical processes. Furthermore, by using Nano-sized MoS_2_ (90–200 nm), we aimed to enhance antimicrobial efficacy while preserving the mechanical and aesthetic properties of GICs.

According to our results, adding 0.05% biogenic Ag NPs to GICs enhanced the antibacterial activity. Furthermore, there was no significant change in antibacterial activity after thermal cycling. Additionally, there was no statistically significant difference in surface microhardness values among the groups or due to thermal cycling. Adding Ag NPs did not result in a clinically significant color change in the GICs. Based on these findings, our study’s first, third, and fourth hypotheses were accepted, while the second hypothesis was rejected.

Conventional glass ionomer cements (GICs) are widely used in dentistry due to their distinctive properties, such as chemical bonding with tooth structures and base metals, fluoride release, thermal compatibility with enamel, biocompatibility, and low cytotoxicity [14]. Research is ongoing to improve the positive characteristics of GICs further. Secondary caries is one of the most common reasons for restoration failure. It is known that biofilm on the surface of restorative materials can facilitate the development of secondary carious lesions [24]. Secondary caries primarily develops due to the invasion of cariogenic bacteria, particularly *S. mutans* [20]. Therefore, the antibacterial activity of restorative materials is crucial in preventing the possibility of secondary caries along the tooth-restoration interface [24]. 

Nanotechnology has been successfully applied in many fields, including dentistry. The development of nanotechnology has led to advances in caries management strategies. Ag NPs are frequently used in various fields, especially in dental practice, due to their superior antibacterial properties. At low concentrations, Ag NPs exhibit significant antibacterial activity against *S. mutans* [25]. The antimicrobial mechanism of Ag NPs is based on the ability of silver ions to inactivate vital bacterial enzymes, leading to cell death [25]. Research has confirmed the antimicrobial efficacy of Ag NPs when incorporated into various restorative materials, such as dental composites, adhesives, and GICs. Ag NPs can exhibit a synergistic antibacterial effect when combined with other antibacterial elements [26]. According to our study’s results, the experimental GIC groups containing Ag NPs and Ag@MoS_2_ NC exhibited significantly higher antibacterial efficacy than the control group, and this effect persisted after thermal aging.

The antibacterial effectiveness observed in GICs modified with silver nanoparticles (Ag NPs) and Ag@MoS₂ nanocomposites (NCs) may be attributed to several interconnected mechanisms, supported by both physicochemical properties of the nanoparticles and the experimental findings of this study. Firstly, the gradual release of Ag⁺ ions is known to disrupt bacterial enzymatic systems, damage cellular membranes, and interfere with DNA replication, which aligns with the observed reductions in *s. mutans* metabolic activity, lactic acid production, and colony-forming units (CFUs) [24]. Secondly, silver-based nanoparticles are capable of inducing the formation of reactive oxygen species (ROS), including hydrogen peroxide and superoxide radicals, which cause oxidative stress and bacterial cell death [24, 25]. Moreover, the MoS₂ nanosheets in Ag@MoS₂ NCs serve as a two-dimensional support material that increases surface area and facilitates the sustained and localized release of silver ions. This nanostructure may also physically hinder bacterial adhesion and biofilm formation through a nano-topographical barrier effect [24, 25]. Notably, the sustained antibacterial activity even after thermal cycling suggests that the nanomaterials remained functionally stable within the GIC matrix. This stability could be attributed to strong physical entrapment and limited leaching from the cement matrix. Lastly, the maintenance of surface microhardness and color stability indicates that the low concentration (0.05%) of nanoparticles was optimized to achieve antimicrobial benefits without compromising the structural or esthetic performance of the GICs.

Different methods are currently used for NP synthesis. Recent studies indicate that NPs produced through green synthesis garner more interest than chemically synthesized NPs [9, 26]. Chemical methods typically require toxic reducing and stabilizing agents, significantly disadvantaging biological applications. Therefore, the demand for non-toxic synthesis methods has increased. The green synthesis method offers a significant advantage in this context.

Additionally, biosynthesis of NPs through green synthesis is a more suitable method than chemical synthesis due to the ease of synthesis and the ability to control the size of NPs [27]. Consequently, biogenic Ag NPs were synthesized in our study using green tea extract. The reasons for choosing green tea include its non-staining properties and antibacterial and antioxidant activity [28]. According to our results, no statistically significant difference was observed in terms of antibacterial activity between the Ag NP groups synthesized by the green synthesis method and the chemical synthesis method. Similarly, no statistically significant difference was observed in terms of antibacterial activity between the Ag@MoS₂ NC groups synthesized by the green synthesis method and the chemical synthesis method.

A study using reduced GO (rGO)/Ag nanocomposites (NC) examined the ability of rGO/Ag to inhibit caries progression. The results indicated that rGO/Ag reduced enamel demineralization, decreased surface roughness, and reduced lesion depth compared to control groups [21]. Similarly, rGO/Ag NC was found to inhibit biofilm formation on conventional GICs and reduce the viability of *S. mutans.* Adding 2% rGO/Ag NC by weight provided antibacterial activity while preserving the mechanical and physical properties of GICs [13]. Moreover, a study incorporating Ag NP and Ag@GO NC into adhesive systems reported successful antibacterial efficacy and bond strength against *s. mutans* in groups with Ag@GO NC. Our study used MoS_2_, a two-dimensional nanocarrier with similar properties to GO. The results showed that experimental GICs containing Ag@MoS_2_ NC synthesized by green and chemical methods exhibited more significant antibacterial activity than those containing Ag NPs alone, regardless of the synthesis method. This increased activity could be due to the homogeneous distribution of Ag NPs on the MoS_2_ surface, which increases the surface area available to interact with bacteria, thereby enhancing antibacterial efficacy [29]. 

Green-synthesized nanoparticles may not exhibit superior antimicrobial activity compared to chemically synthesized ones due to several factors. Plant extracts can coat nanoparticle surfaces with phytochemicals, hindering direct contact with bacterial membranes and limiting ion release, thereby reducing effectiveness. Additionally, the low colloidal stability of green-synthesized nanoparticles can lead to aggregation, decreasing surface area and antimicrobial activity. Therefore, the performance of green-synthesized nanoparticles may fall short of expectations due to plant matrix effects and variations in synthesis conditions, indicating the need for careful optimization of synthesis parameters for more consistent results [30, 31]. 

Advancing technology and changing patient expectations have led to the adoption of aesthetic approaches in dentistry [14]. Due to their dark gray color, Ag NPs can alter the color of the materials they add. Therefore, Ag NPs should be used in low quantities to avoid negatively impacting dental materials’ color, aesthetics, and mechanical properties. Studies have shown that appropriately dosed Ag NPs significantly reduce biofilm growth and lactic acid production while providing strong antibacterial activity without negatively affecting dental materials’ aesthetic, physical, and mechanical properties [32]. Consequently, among the materials developed using nanotechnology, the goal is to achieve the best results using the lowest concentration of Ag NPs. Our study determined that 0.05% was the most effective minimum amount for Ag NPs to limit adverse effects in this context. Additionally, the color of GIC was measured initially, after NP addition, and after thermal cycling, and the ΔE values were calculated. The calculated ΔE values indicated no clinically significant color change.

Another parameter determining the longevity of dental restorative materials is their mechanical properties. Therefore, adding Ag NP and Ag@MoS_2_ NC to the experimental GIC is expected not to affect the material’s mechanical structure adversely. Among the mechanical parameters, microhardness is also examined. Microhardness is the resistance to forming permanent indentations on the material’s surface. Low hardness values are typically associated with low wear resistance and increased susceptibility to scratching [14]. In our study, adding Ag NP and Ag@MoS_2_ NC to GICs did not alter the microhardness values. Furthermore, no statistically significant change was observed in microhardness values after thermal cycling. These results may be due to using NPs at an effective minimum concentration.

The novel aspect of our study lies in incorporating Ag NPs synthesized by green synthesis methods and Ag@MoS_2_ NC into GICs and analyzing the impact on antibacterial activity, color stability, and mechanical properties. Our study’s limitations include using only 0.05% Ag NP, using only MoS_2_ as a nanocarrier, and the absence of evaluating parameters like surface microhardness test. The antimicrobial limitation of our study is that oral conditions and bacterial diversity cannot be simulated in microbiological tests. Although *S. mutans* is a primary contributor to caries development, the oral biofilm comprises various microbial species that can act synergistically. Therefore, the antimicrobial results observed in this study may not fully reflect anti-plaque or anti-caries efficacy under clinical conditions. In future studies, it may be beneficial to analyze the physical and mechanical properties by incorporating different Ag NP ratios and nanocarriers into the same material.

## Conclusion

The following conclusions can be drawn within the limitations of the study:

- Both green and chemical synthesis methods produced Ag NPs and Ag@MoS_2_ NC, providing adequate antimicrobial activity in the experimental GICs.
- Ag NPs and Ag@MoS_2_ NC, regardless of whether obtained via green or chemical synthesis methods, did not adversely affect the microhardness of the experimental GICs.
- Ag NPs and Ag@MoS_2_ NC, produced through both synthesis methods, did not result in a clinically significant color change in the experimental GICs. In order to obtain more detailed results on this subject, further studies may be considered to test Ag NP concentrations, use alternative carriers, and evaluate the mechanical properties and biocompatibility of the material.

## Data Availability

No datasets were generated or analysed during the current study.
